# Structural design principles for specific ultra-high affinity interactions between colicins/pyocins and immunity proteins

**DOI:** 10.1038/s41598-021-83265-2

**Published:** 2021-02-15

**Authors:** Avital Shushan, Mickey Kosloff

**Affiliations:** The Department of Human Biology, Faculty of Natural Sciences, University of Haifa, 199 Aba Khoushy Ave., Mt. Carmel, 3498838 Haifa, Israel

**Keywords:** Proteins, Biochemistry, Structural biology, Protein analysis, Computational biology and bioinformatics, Protein design, Antimicrobials

## Abstract

The interactions of the antibiotic proteins colicins/pyocins with immunity proteins is a seminal model system for studying protein–protein interactions and specificity. Yet, a precise and quantitative determination of which structural elements and residues determine their binding affinity and specificity is still lacking. Here, we used comparative structure-based energy calculations to map residues that substantially contribute to interactions across native and engineered complexes of colicins/pyocins and immunity proteins. We show that the immunity protein α1–α2 motif is a unique structurally-dissimilar element that restricts interaction specificity towards all colicins/pyocins, in both engineered and native complexes. This motif combines with a diverse and extensive array of electrostatic/polar interactions that enable the exquisite specificity that characterizes these interactions while achieving ultra-high affinity. Surprisingly, the divergence of these contributing colicin residues is reciprocal to residue conservation in immunity proteins. The structurally-dissimilar immunity protein α1–α2 motif is recognized by divergent colicins similarly, while the conserved immunity protein α3 helix interacts with diverse colicin residues. Electrostatics thus plays a key role in setting interaction specificity across all colicins and immunity proteins. Our analysis and resulting residue-level maps illuminate the molecular basis for these protein–protein interactions, with implications for drug development and rational engineering of these interfaces.

## Introduction

The interactions of the antibiotic proteins colicins with immunity proteins has been a seminal model system in numerous studies of protein–protein interactions and specificity (reviewed in Ref.^1–6^). Colicins are produced by *Escherichia coli* strains in high-affinity complexes with their cognate immunity proteins, which inhibit the cytotoxic activity of colicins in the bacteria that produce them^1,5,7–9^. Similar to colicins, pyocins are also antibiotic proteins that are produced by *Pseudomonas* bacteria, including the clinically relevant pathogen *Pseudomonas aeruginosa*^10–14^. Colicins and pyocins have important clinical implications, as they have been shown to function as virulence factors and are considered promising candidates for protein-based antibiotics^15–23^. The binding of colicins/pyocins to their cognate immunity proteins is mediated by the cytotoxic domains of the former and is characterized by ultra-high (up to femtomolar) affinities; non-cognate pairs from different bacteria bind with weaker affinities that are 6–10 orders of magnitude lower, but are nevertheless easily measurable^24–30^. However, a complete understanding of how cognate vs. non-cognate interactions are set at the individual residue-level remains to be fully elucidated.

When comparing different colicins and pyocins, previous studies have used two alternative classification schemes based on dissimilar criteria. One scheme classified colicins according to mechanisms of entry into the target cell^5,9,31–34^, while the second scheme classified colicins according to their cytotoxic mechanism, i.e., DNases, RNases, tRNases, and pore forming colicins^1,5,9^. For example, colicins E2, E7, E8, and E9 are DNases while colicins E3, E4, and E6 are RNases; yet, all of these colicins bind to the same receptor in the target cell. Previous structural studies of DNase colicins have shown that colicins E2, E7, and E9 share a global structural similarity, as do their corresponding immunity proteins^35–37^. Interestingly, the pyocins Pyo-S2 and Pyo-AP41 are also DNases and their structures were shown to be similar to the DNase colicin Col-E2^14^. On the other hand, while the cytotoxic domains of the tRNase colicins Col-E5 and Col-D were shown to share the same 3D fold, their active sites differ, and they were suggested to differ from the cytotoxic domains of colicins E2, E7, and E9^1,5^. Nonetheless, a comprehensive structural comparison between all of these colicins and pyocins in the context of their interface with their cognate immunity proteins has not been performed.

Previous structural studies have suggested that the majority of residues involved in interactions between colicin/pyocin DNases and their immunity proteins are located in the α4 helix and the following α4–β2 loop of colicins/pyocins, and in the region encompassing the α2 and α3 helices of immunity proteins^8,14,36–38^. The central importance of the immunity protein α2–α3 region for binding their cognate colicin partners has been demonstrated in numerous mutagenesis and computational studies of immunity proteins Im2 and Im9^26,30,37,39,40^. These studies have pinpointed 9 to 11 residues in the α2–α3 region of immunity proteins but only two to three residues in the α1–α2 and α3–α4 loops that, upon mutation, affected colicin binding. On the other side of the interface (i.e., colicins), a combined computational and biochemical analysis of Col-E9 showed that four residues in the α4 and α4–β2 loops and two residues in the α2 helix and the α3–α4 loop were important for binding Im9^29^. Studies of the non-cognate complexes of Col-E2–Im9 and Col-E9–Im2 have suggested that the immunity protein α3 helix functions as a conserved binding motif across different immunity proteins, whereas the α2 helix determines specificity^26,27,29,30,37,41,42^. Several of these studies showed that alanine mutagenesis in the α3 helix of Im2 and Im9 reduced affinity in both cognate and non-cognate complexes similarly—supporting the role of the α3 helix as a common and conserved anchor for binding colicins. On the other hand, replacement of the Im2 α2 helix with its Im9 counterpart increased affinity to Col-E9 to the level of the cognate complex^41^, supporting a suggested role of the α2 helix as a specificity determinant. Subsequent studies mutated three to six residues in the α2 helix of Im2, Im7, and Im9, further supporting this hypothesis^26,27,40,42,43^. On the other side of the interface, replacement of six residues in the α4–β2 loop of Col-E9 with the corresponding residues from Col-E8 reduced interactions with Im9, suggesting this region determines the specific interactions of Col-E9 with its cognate partner^44^. A later study mutated additional residues in the Col-E9 α4–β2 loop and showed a stronger reduction in binding to Im9 compared to Im2, highlighting the importance of this region in determining specificity^29^. Nevertheless, a precise and quantitative definition of which structural elements and residues in DNase colicins/pyocins and their immunity proteins contribute to binding affinity and to partner specificity is still lacking.

The colicin-immunity protein system has also been used as a model system for computational design of protein–protein interactions and in vitro evolution of interaction specificity^45–48^. Studies employing computational redesign of the Col-E7–Im7 complex have shown that replacement of 1 to 2 residues in the α2 helix and/or the α2–α3 loop of Im7 and 2 to 4 residues in the α4–β2 region of Col-E7 were sufficient to achieve Col-E7–Im7 pairs that bound each other better than either of their wild-type counterparts^45,46^. Using in vitro evolution, Levin et al*.* showed for the first time that changes in the α1–α2 loop of Im9 were necessary to evolve an immunity protein to bind a non-cognate partner with ultra-high affinity^47^. While this was the first mention of the α1–α2 loop as affecting interactions with colicins, this result was interpreted as an indirect effect mediated through conformational changes, as the residues in this loop were deemed non-contacting residues. However, in a recent study that designed novel high-affinity binders of Col-E2, the entire α1–α2 loop of Im2 needed to be replaced with artificial loops, further highlighting a role for this immunity protein loop in reaching high affinity towards colicins^48^. Clearly, a comprehensive framework that maps all interaction and specificity determinants across colicins/pyocins and their interactions with immunity proteins can guide future engineering and design of these systems.

On the other hand, colicins E3, E5, and D are structurally different from the DNase colicins and from each other, as are their immunity proteins^1,5,49–52^. Uniquely, the structure of Col-E3 with Im3 showed two separate interfaces: one with the Im3 cytotoxic domain, which is analogous to available structures of other colicins/immunity proteins complexes, and a second Im3 interface with the Col-E3 translocation domain, which is not represented in other structures^49^. Mutagenesis studies implicated one residue in the β3 strand of Im3^53^, eight residues in the α2 and α3 helices of ImD^51^, and four residues in the α3 helix, β1 strand, and the N-terminus of Im5^54^, as crucial residues for binding their cognate colicins. However, comprehensive information on which residues are important for binding in the Col-E3–Im3, Col-E5–Im5, and Col-D–ImD complexes is lacking.

Here, we present a comprehensive computational study that compares representative 3D structures from all available colicin/pyocin–immunity protein complexes. We used structure-based energy calculations to accurately identify the residues that substantially contribute to the interactions in all of these structures, pinpointing both general and specific determinants to inter-molecular interactions. Our structure analysis and energy-based residue-level maps provide a better understanding of the molecular basis for protein–protein interactions between colicins/pyocins and immunity proteins—as a model system for protein–protein interactions with clinical implications for drug development, and as a detailed map that can guide future engineering of these interfaces.

## Results

### Colicins/pyocins and their cognate immunity proteins can be classified into four families based on structural similarity

**Figure 1:**
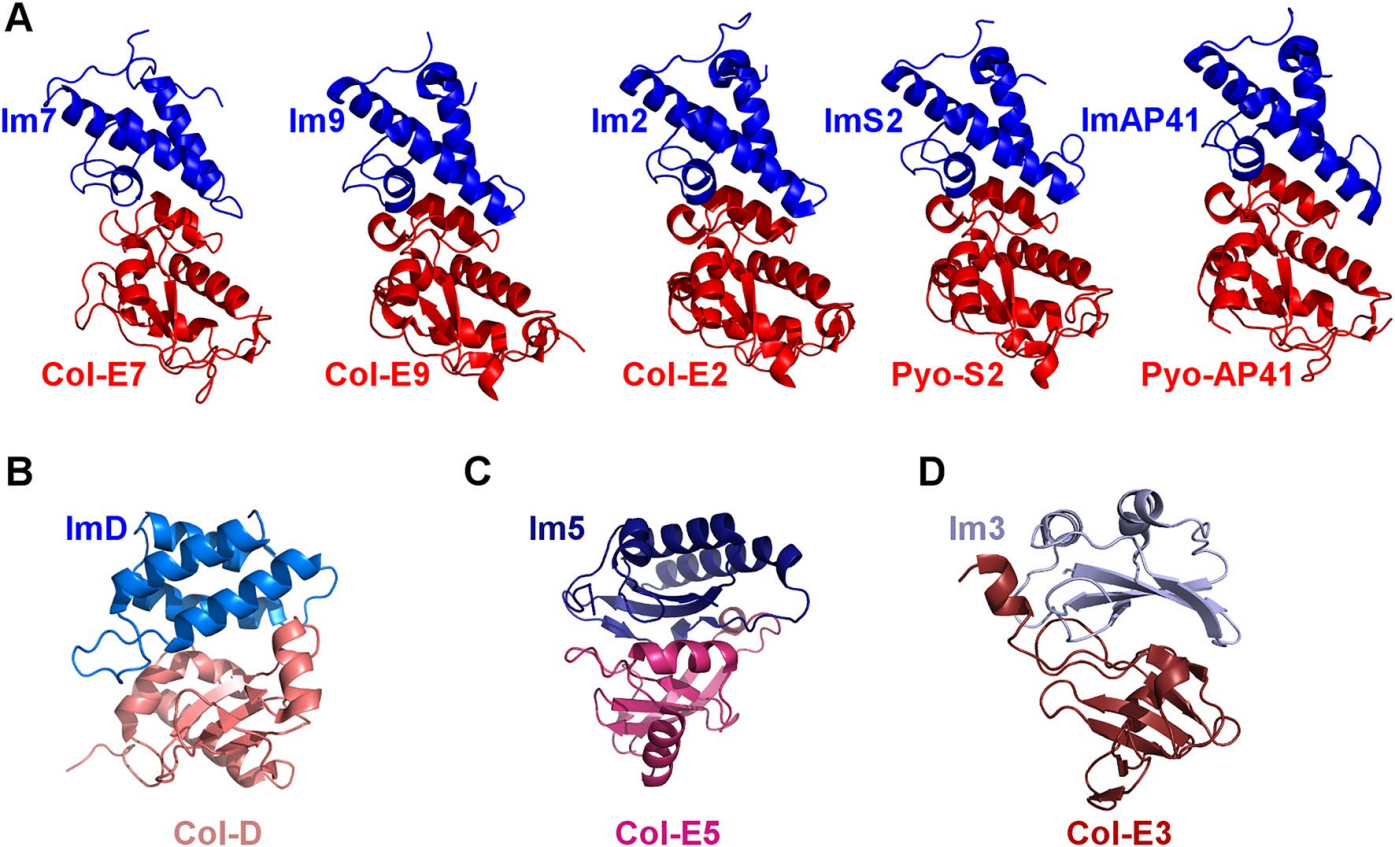
Colicins/pyocins and their cognate immunity proteins can be classified into four families based on structural similarity. Structures of different colicin/pyocin–immunity protein complexes (with representative PDB IDs), shown as ribbon diagrams (colored in shades of red and blue, respectively), can be divided into four families based on structure alignment. (**A**) The E7/Im7-like family: Col-E9–Im9 (1EMV), Col-E2–Im2 (3U43), Col-E7–Im7 (7CEI), Pyo-S2–ImS2 (4QKO), and Pyo-AP41–ImAP41 (4UHP). (**B**) The Col-D/ImD family (1V74). (**C**) The Col-E5/Im5 family (2FHZ). (**D**) The Col-E3/Im3 family (2B5U).

In order to compare the interactions between different colicins/pyocins and their cognate immunity proteins, we first classified available structures into families according to structural similarity—comparing eight representative experimentally-solved complexes of colicins/pyocins–immunity proteins^14,36–38,49,51,54^—see “Materials and methods” section for details. This classification ensured we group all comparable protein complexes together before performing a residue-level analysis. Structural alignments of the E2, E7, and E9 colicins and the S2 and AP41 pyocins bound to their cognate immunity proteins showed that the structures of these monomers, as well as the quaternary structure of the complexes, are highly similar (Fig. 1A). The sequence identify among the immunity proteins was ~ 50–70% while the sequence identity among these DNase domains was ~ 50–80%. The Root Mean Square Deviation (RMSD) of the structural alignments of colicins and of immunity proteins ranged between 1 and 1.5 Å for more than 90% of the full length of the structures, respectively, as expected from previous structural analyses^14,35–37^. We therefore grouped these immunity proteins–colicins together and termed them the “Im7-like family” and the “E7-like family”, respectively. This grouping corresponds to colicin classification by mechanism of cytotoxicity, since all E7-like family members are DNases^1,5,9^. Furthermore, Joshi et al*.* compared these proteins to 12 more DNase-Im pairs, showing all 17 pairs shared sequence identities above 30% and 40%, suggesting all of these proteins belong to the same structural group^14^. On the other hand, the structures of colicins D, E5, and E3 and their cognate immunity proteins were structurally dissimilar—both among themselves and when compared to the structures of the E7/Im7-like families (Fig. 1). Comparing ImD to Im7 and Col-E3 to Col-E5, we observed aligned regions of only 24 and 25 residues, respectively, with sequence identities of ~ 5–10%. However, these aligned substructures interacted with structurally non-related segments of their cognate partners and the interfaces showed no comparable similarities. We therefore concluded that each of these structures represents a distinct structural family (Fig. 1B–D).

### The immunity protein α1–α2 motif adopts dissimilar conformations in most Im7-like family members

**Figure 2:**
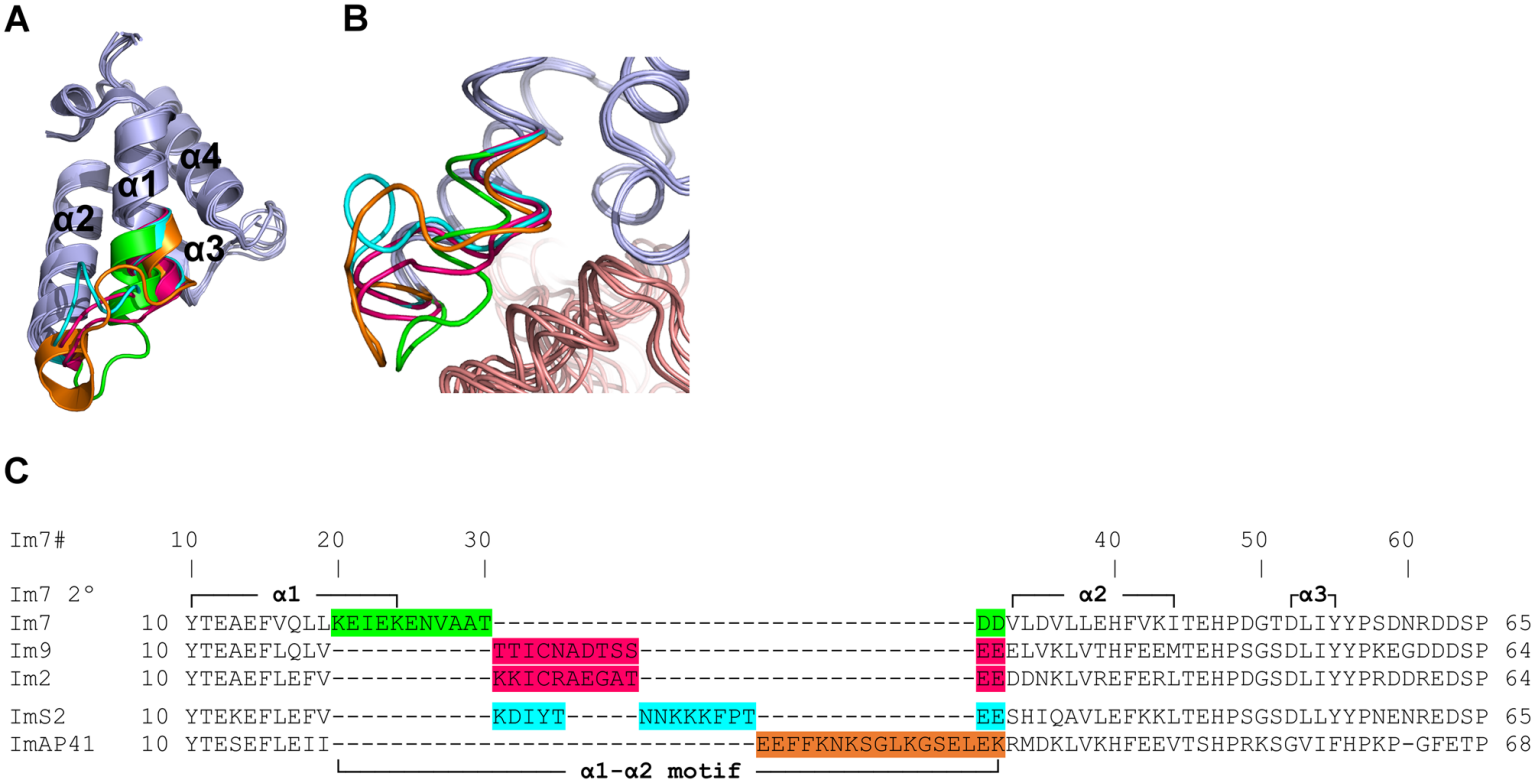
The α1–α2 loop and adjecent residues in immunity proteins adopt different conformations in members of the Im7-like family. (**A**) The immunity protein “α1–α2 motif”, defined as the structurally-dissimilar region with observed Cα distances of more than 1.5 Å between at least two of the structures. The representative structures from the Im7-like family (Fig. 1A) were superimposed and their α1–α2 motifs were colored as follows: Im7, green; Im9 and Im2, pink; ImS2, cyan; ImAP41, orange. (**B**) Close-up of the α1–α2 motif, rotated 45° about the Y-axis relative to (**A**). Colicins and immunity proteins are colored pink and light blue, respectively. (**C**) Structure-based sequence alignment of the Im7-like family. The structurally-dissimilar α1–α2 motifs are color-coded as in A. Im7 secondary structure elements are marked above and below the alignment. Note that only the Im9 and Im2 α1–α2 motifs were structurally-similar.

The structural alignments performed above show that the α1–α2 loop region in the Im7-like family is the only immunity protein sub-structure located at the interface with colicins/pyocins that was structurally-dissimilar among the Im7-like family. We defined the extent of this dissimilar region to include those residues with observed Cα distances of more than 1.5 Å in at least two of the structures. We termed this structurally-dissimilar region the “α1–α2 motif”, as it extends beyond the α1–α2 loop—it encompasses residues that correspond to positions Im7#20–30 (Fig. 2). The regions surrounding the α1–α2 motif were structurally similar in all five structures, adopting a highly similar 3D conformation (Fig. 2A). The α1–α2 motifs in Im7 and ImAP41 were especially unique in their structures, showing a dramatically different conformation than all other immunity proteins. In ImS2, the N-terminal segment of the α1–α2 motif, which is the C-terminus of the α1 helix, is structurally similar to Im9 and Im2, while the C-terminal segment of the ImS2 α1–α2 motif is structurally divergent (Fig. 2B,C). On the other hand, there were only two immunity proteins that were structurally-similar across the entire α1–α2 motif–Im9 and Im2—a noteworthy observation given that most previous studies of non-cognate complexes investigated interactions between Im9/Im2 and Col-E9/Col-E2^26–30,41,42,48^.

### Residue-level mapping of the interactions of colicins with their cognate immunity proteins

**Figure 3:**
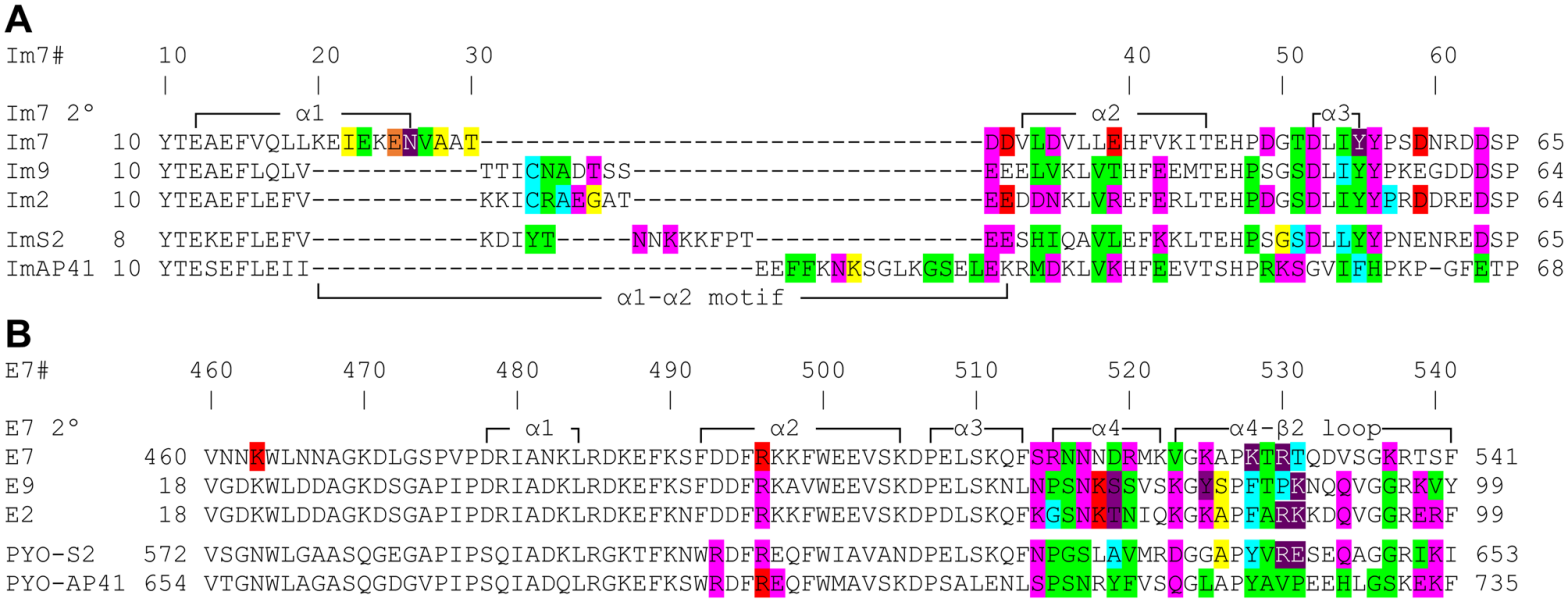
Residues in colicin–immunity protein complexes that are predicted to contribute to inter-molecular interactions in the E7-like family and the Im7-like family. (**A**,**B**) Residue-level sequence maps summarizing the structure-based energy calculations for the complexes of immunity proteins (**A**) with their cognate colicins/pyocins (**B**). Residues that contribute substantially to interactions are colored according to the type of their energy contribution: non-polar contributions (np), green; side-chain electrostatic contributions (sc elec), red; main-chain electrostatic contributions (mc elec), yellow; sc elec and mc elec, orange; sc elec and np, magenta; mc elec and np, cyan; sc elec, mc elec and np, purple. Immunity protein and colicin/pyocin positions are numbered according Im7 (Im7#) and Col-E7 (E7#) and together with the Im7/Col-E7 secondary structure elements are shown above and below the alignment.

**Figure 4:**
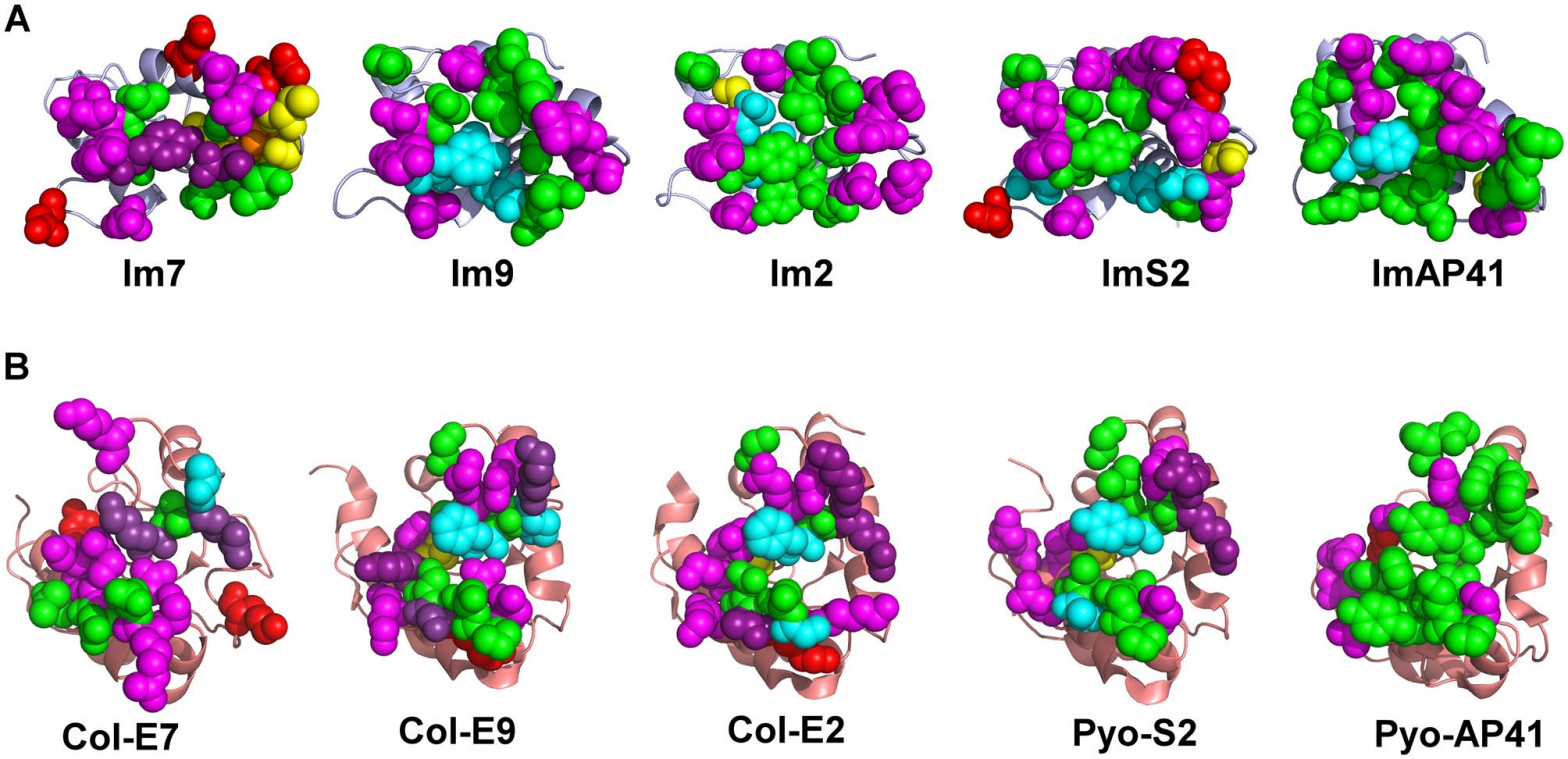
3D visualization of residues that contribute substantially to interactions between the Im7-like family and the E7-like family. (**A**) Residues in the Im7-like family that substantially contribute to interactions with their cognate colicin/pyocin partners, as in Fig. 3A. (**B**) Residues in the E7-like family that substantially contribute to interactions with their cognate partners, as in Fig. 3B. Substantially contributing residues are shown as spheres and colored as in Fig. 3. Immunity proteins and colicins/pyocins are shown in an “open book” view, where the immunity proteins and colicins/pyocins are rotated ~ 90° about the X-axis in oppositve directions relative to Fig. 1A and shown as ribbons colored light blue and salmon, respectively.

To map the individual residues that contribute to colicin/pyocin–immunity protein interactions, we analyzed the representative X-ray structures of colicins bound to their immunity proteins, using an energy-based computational methodology developed previously by our lab^55–61^. As described in the “Materials and methods” sectio, we calculate the net electrostatic/polar contributions (ΔΔG_elec_) of each residue ≤ 15 Å of the colicin–immunity protein interfaces. The non-polar energy contribution (ΔΔG_np_) of each residue was calculated separately, based on residue burial in the complex relative to the unbound monomers. Note that since the electrostatic calculations output the net difference between the intermolecular interactions of a residue with a cognate protein partner, relative to the interactions with the water and ions in the solvent, they identify only residues that are predicted to contribute to protein–protein binding substantially. To reduce false positives and negatives, we applied a consensus approach that compares biological replicates across multiple dimers in an asymmetric unit or PDB structures (see “Materials and methods” section, Supplementary Fig. S1), substantially improving the accuracy of the predictions. Residues thus calculated to contribute substantially to intermolecular interactions (Figs. S2, S3) were mapped to the sequence (Fig. 3) or the structure of each individual protein (Fig. 4).

Our calculations showed that the number of colicin/immunity protein residues that substantially contribute to intermolecular interactions is similar across the family, usually ranging from 15 to 20 residues, with the smallest number of contributing residues (15 residues) found in Col-E7 (Fig. 3). Most colicin/pyocin residues that substantially contribute to binding are located in the α4–β2 loop, but also in the preceding α4 helix. Most of the immunity protein residues that substantially contribute to interactions with colicins are located in the α2 and α3 helices, but about a third of the contributing residues are located in the preceding α1–α2 motif.

Strikingly, the majority of the contributions from colicins and immunity protein residues to interactions with their cognate partners involve electrostatic contributions. Some of these electrostatic interactions are long-range (> 5 Å), mediated by the following residues that are distant from their cognate partners: Im7–Asp32/Asp59 and Col-E7–Lys463/Arg496, Col-E9–Lys76, and Col-E2–Lys76. However, the ImAP41–Pyo-AP41 complex stands out in having more non-polar only interactions across the interface, which also leads to a prediction that this interaction will be less affected by changes in salt concentration. In a global view, non-polar contributions were similar across different colicin/pyocin–immunity protein complexes (Figs. 3, S2–S4), likely because of the general structural similarity of the interfaces across the five complexes of the E7/Im7-like families (Figs. 1, 4, S4). The main reason for differences in energy contributions among conserved residues across immunity proteins is that the residues in the partner colicin/pyocin that interact with these conserved residues are different. Taken together, our results suggest that particular electrostatic interactions may play a critical role in determining specificity between colicins/pyocins and their cognate immunity proteins.

### Particular electrostatic contributions mediate specific interactions between immunity proteins and colicins/pyocins

To investigate how contributing residues interact across the interfaces, and in particular those residues that contribute via electrostatic interactions, we examined which colicin/pyocin residues interact with the following three structural regions in immunity proteins: the Im α1–α2 motif, the α2 helix, and the following extended loop region that includes the short α3 helix (the “α3 region”). For each of these structural regions, we found a different interaction scheme across the interface with the cognate colicin/pyocin.

**Figure 5:**
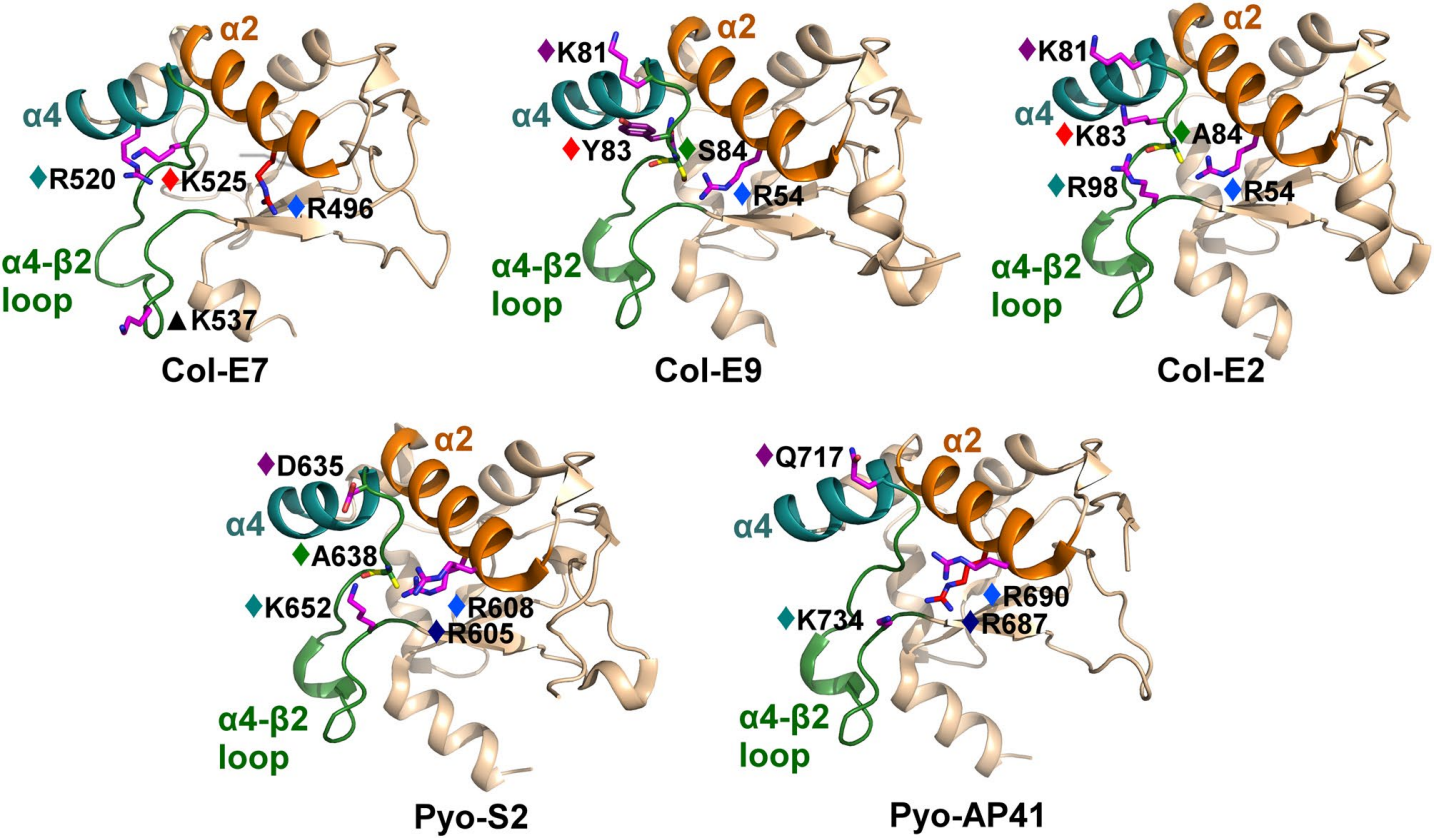
Similar electrostatic contributions of colicin/pyocin residues to interactions with the divergent Im α1–α2 motif. Colicin residues that substantially contribute electrostatically to interactions with the Im α1–α2 motif are shown as sticks, colored according to their energy contributions, as in Fig. 3. Colicin residues that contribute similarly to interactions across multiple members are marked with diamonds, colored as follows (with the corresponding E7 position numbering as in Fig. 3B): dark blue (E7#493), light blue (E7#496), teal (E7#520 or E7#540, see also Supplementary Fig. S5), purple (E7#523), red (E7#525), and green (E7#526). Colicin residues that contribute to interactions with the cognate immunity protein only in one complex are marked with black triangles. The colicin/pyocin α2 helices are colored orange, the α4 helices are colored cyan, and the α4–β2 loops are colored green.

We observed a surprising conservation of electrostatic contributions among the colicin/pyocin residues that interact with the Im α1–α2 motif (Fig. 5), despite its sequence and structure dissimilarity (Fig. 2) and the variable contributions from different residues across these Im motifs (Fig. 3A). Between four to five residues in each structure, originating from six colicin/pyocin positions, contribute to interactions with the Im α1–α2 motif; these six positions are located across the colicin/pyocin α2 and α4 helices and in the α4–β2 loop. The electrostatic contributions coming from these positions are similar in most or all complexes, although the contributing residues themselves are not necessarily conserved. In particular, a prominent interaction across all complexes is observed between one to three positively-charged colicin/pyocin residues in positions E7#493 and E7#496 and in position E7#520 or position E7#540 that together converge to an electrostatic interaction with a single negatively charged residue at a conserved position in the C-terminus of the Im α1–α2 motif (position Im7#31, Supplementary Fig. S5). A colicin-specific electrostatic contribution from residues in the E7#525 position is conserved across all three colicins, but does not have a corresponding contribution in pyocins, while the contributions from positions E7#523 and E7#526 are observed in four and three colicins/pyocins, respectively.

**Figure 6:**
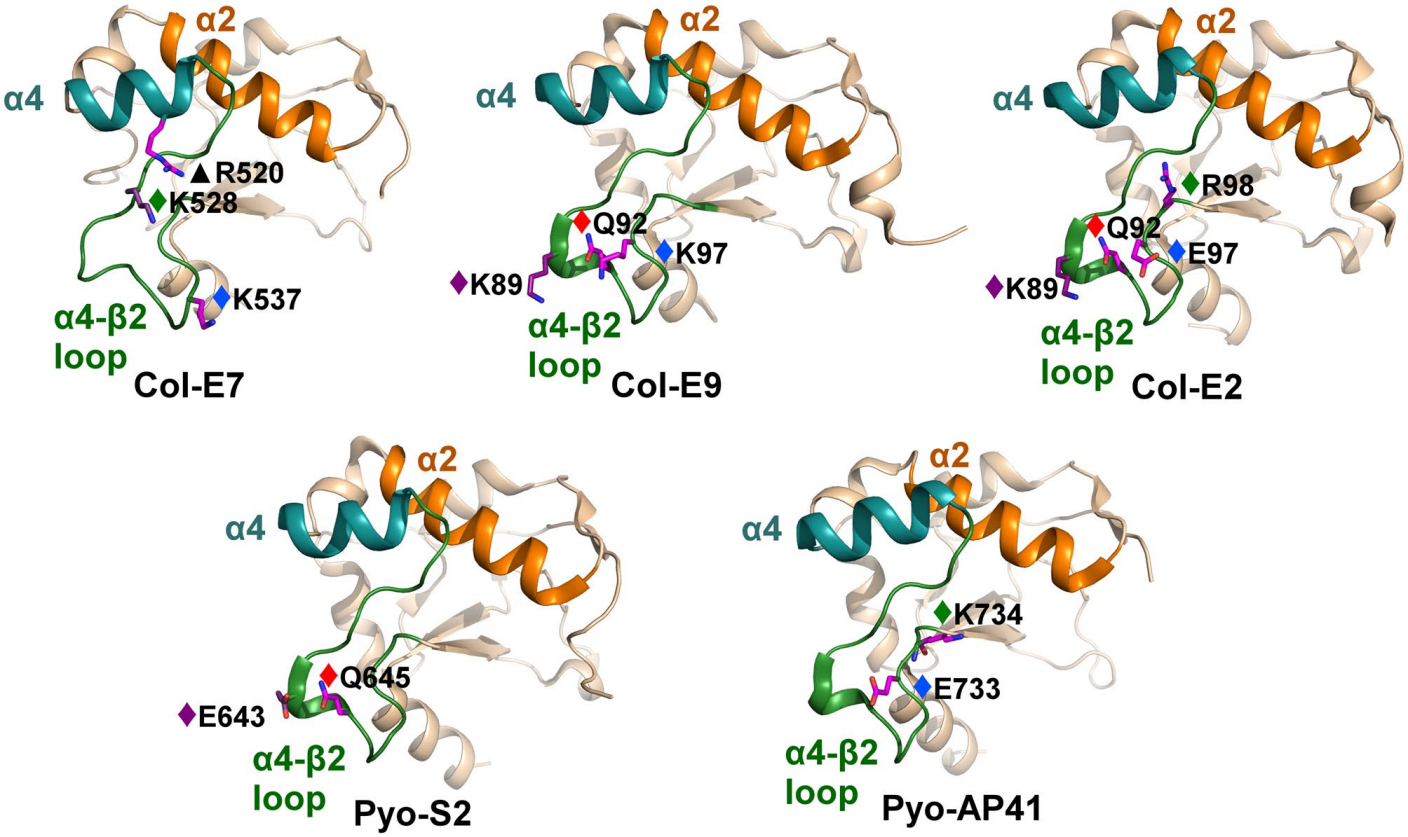
Limited electrostatic contributions of colicin/pyocin residues to interactions with the α2 helix and adjacent residues in immunity proteins. Colicin residues that substantially contribute electrostatically to interactions with the Im α2 helix are shown as sticks and colored according to their energy contributions, as in Fig. 5. Colicin residues that contribute similarly to interactions across multiple members are marked with diamonds and colored as follows (the corresponding E7 position number, as in Fig. 3B, is also noted): purple (E7#531), red (E7#534), light blue (E7#539), and green (E7#528 or E7#540). Colicin residues that contribute to interactions with the cognate immunity protein only in one complex are marked with black triangles. The colicin/pyocin α2 helices are colored orange, the α4 helices are colored teal, and the α4–β2 loops are colored green. Colicin/pyocin structures are rotated 35° about the Y-axis relative to Fig. 5.

The electrostatic contributions of colicins/pyocins to interactions with the Im α2 helix were more limited—two to four contributing residues (Fig. 6). Almost all contributions to interactions with this Im helix originated from the C-terminal part of the colicin/pyocin α4-β2 loop. Only in Col-E7 did we observe a single contribution from the α4 helix. The contributions from Col-E9 and Col-E2 were especially similar, but contributions from the two pyocins were also similar to two Col-E9/Col-E2 contributions. Here, we also observed a positively-charged residue that converges from Col-E7, Col-E2, and Pyo-AP41 (E7#528 or E7#540, Fig. 6) to contribute similarly to electrostatic interactions with a single aspartate/asparagine residue in the Im7 α2 helix (Im7#35, Supplementary Fig. S6A). A similar electrostatic interaction also occurs between a lysine in Col-E9 and Col-E2 (position E7#531) and a glutamate in the corresponding Im α2 helix (Im7#42, Supplementary Fig. S6B). Interestingly, in Pyo-S2 we see a reciprocal salt bridge to this interaction—between a negatively-charged glutamate in the pyocin (E7#531) and a positively-charged residue in the immunity protein (Im7#42, Supplementary Fig. S6B).

**Figure 7:**
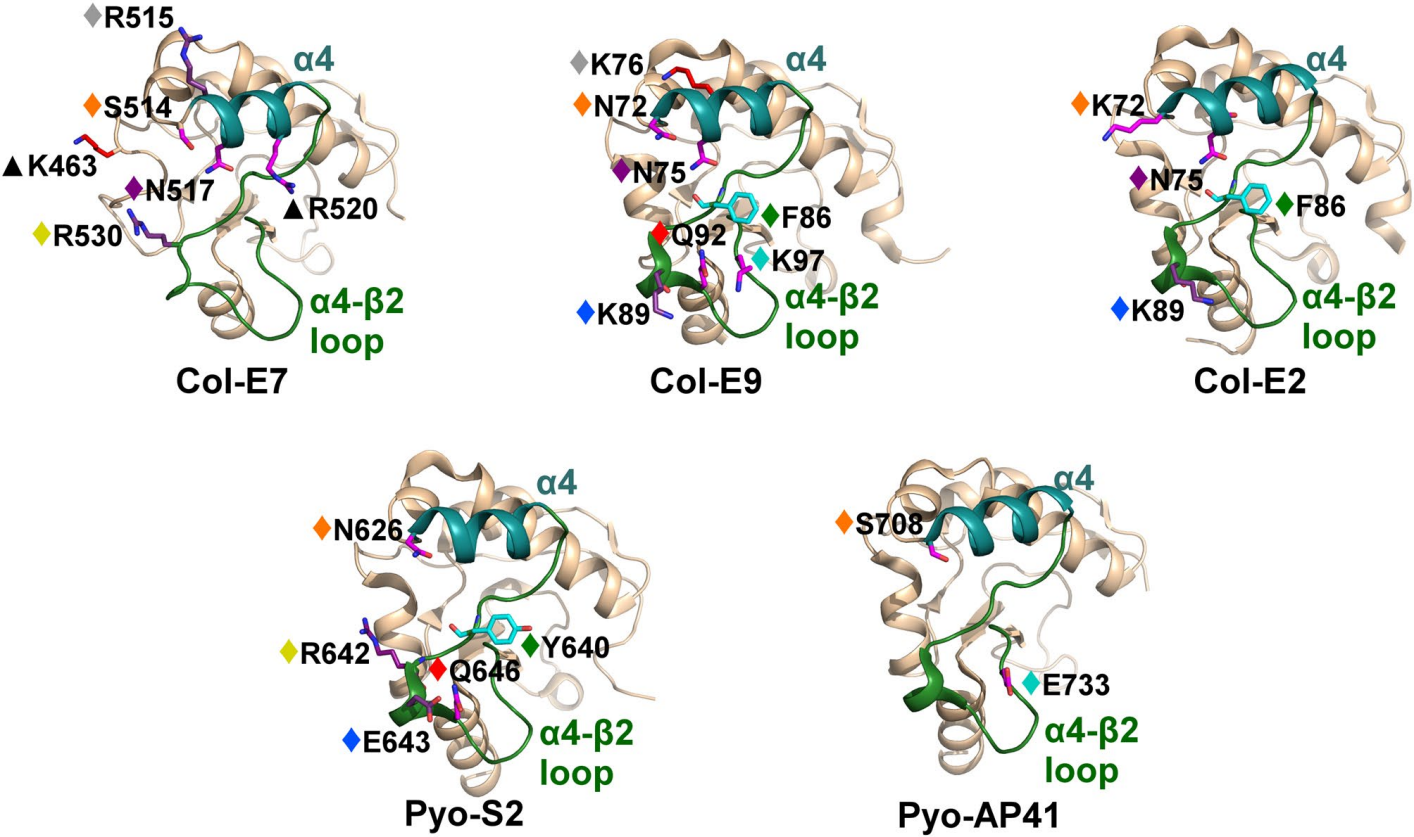
Varied electrostatic contributions of colicin/pyocin residues to interactions with the Im α3 region. Colicin residues that substantially contribute electrostatically to interactions with the Im α3 helix are shown as sticks, colored according to their energy contributions, as in Fig. 3. Colicin residues that contributed similarly to interactions across multiple members are marked with diamonds, colored as follows (the corresponding E7 position number, as in Fig. 3B, is also noted): orange (E7#514), gray (E7#515), purple (E7#517), green (E7#528), yellow (E7#530), blue (E7#531), red (E7#534), and cyan (E7#539). The α4 helices are colored cyan and the α4–β2 loops are colored green. Residues that contributed to interactions with the cognate immunity protein only in one complex are marked with black triangles. Colicin/pyocin structures are rotated 70° about the Y-axis relative to Fig. 5.

Interestingly, the electrostatic contributions of colicins/pyocins to interactions with the Im α3 region were more diverse (Fig. 7), despite the higher sequence conservation of this region. These divergent electrostatic contributions originated mainly from the colicin/pyocin α4 helix and α4–β2 loop. While one colicin/pyocin position seems to contribute similarly to interactions with the Im α3 region across all colicin/pyocin structures (E7#514), the interactions of the residues in this position actually vary considerably among the structures (Supplementary Fig. S7). In most of these colicin/pyocin positions (E7#517, E7#528, E7#530, E7#531, E7#534, E7#539, and E7#540) we observed similar contributions across only three of the five structures (Fig. 7), but not always the same three structures. For example, the asparagine residue in position E7#517 interacted similarly with a tyrosine in position Im7#56 across the three colicins, while the residues in the E7#528 and E7#531 positions contributed similarly in Col-E9, Col-E2, and Pyo-S2. Note that the residues in the E7#531 position also interact with the Im α2 helix (Supplementary Fig. S6). Three contributions were unique to Col-E7 (E7#463, E7#515, and E7#520). Overall, each colicin/pyocin has a different pattern of electrostatic contributions that converge to interact with the similar Im α3 region.

### Comparison of engineered proteins from the E7/Im7-like families to their wild-type counterparts

**Figure 8:**
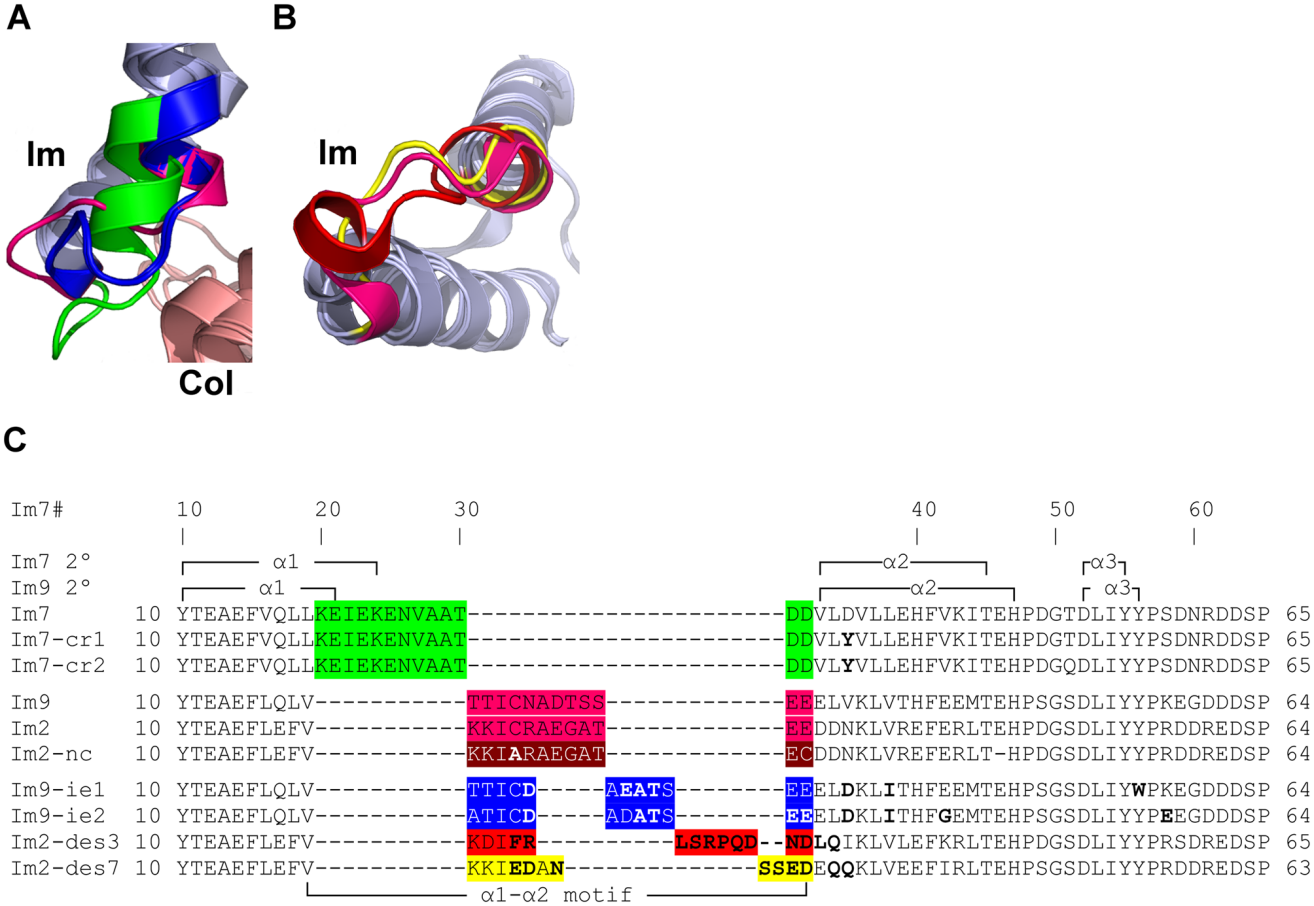
Dissimilar conformations of the α1–α2 motif in the immunity proteins of engineered Im7-like family structures. (**A**) The α1–α2 motifs in the two in vitro evolved immunity proteins (Im9-ie1, Im9-ie2) adopted the same unique conformation. The α1–α2 motifs are colored as follows: Im7, green; Im9, magenta, Im9-ie1 and Im9-ie2, blue. Colicins and immunity proteins are colored pink and light blue, respectively. (**B**) The α1–α2 motifs of the designed immunity proteins (Im2-des3, a “specific” design for Col-des3; Im2-des7, a “multi-specific” variant that recognizes three different designed colicins) adopt different conformations than in Im2. The angle of view is rotated 70° about the X-axis relative to A. The α1–α2 motifs of Im2, Im2-des3, and Im2-des7 are colored magenta, red, and yellow, respectively. (**C**) Structure-based sequence alignment of wild-type and engineered immunity proteins. The α1–α2 motifs are colored as follows: Im7, green; Im9 and Im2, pink; Im2 of a non-cognate pair (Im2-nc), brown; Im9-ie1 and Im9-ie2; blue; Im-des3, red; Im-des7, yellow. Mutations that were introduced into the engineered immunity proteins are marked in bold. Immunity protein positions are numbered according to Im7 (Im7#), shown with the Im7 and the Im9 secondary structure elements above and below the alignment.

We examined how the interactions among non-native pairs of colicins and immunity proteins compare to the interactions between their wild-type counterparts, which were analyzed above. These non-native pairs include the non-cognate Col-E9–Im2 complex^30^ and proteins engineered using computational redesign^45,46,48^ and/or in vitro evolution^47,48^—mostly modifying the immunity protein side of the interface. In the non-cognate Col-E9–Im2 complex and in the two computationally redesigned Col-E7–Im7 complexes the Im α1–α2 motif had the same conformation as the wild-type counterpart (data not shown). On the other hand, in all other non-native pairs at least part of the α1–α2 motif adopted a different conformation than in the wild-type structures (Fig. 8).

**Figure 9:**
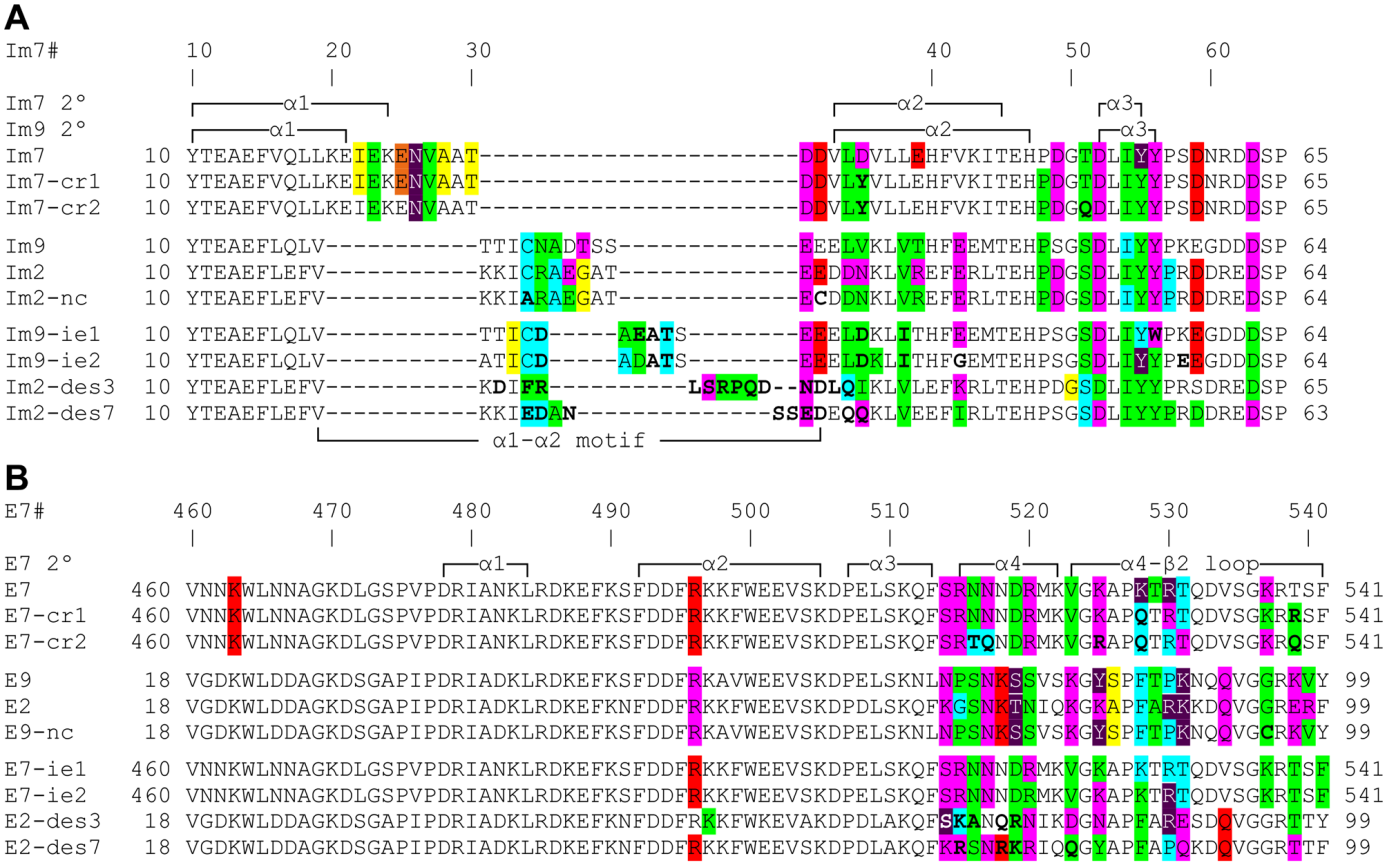
Residues in engineered colicin–immunity protein compexes that are predicted to contribute to inter-molecular interactions in the Im7-like family and E7-like family. (**A**,**B**) Residue-level sequence maps summarizing our structure-based energy calculations for the complexes of engineered immunity proteins (**A**) with their engineered colicins/pyocins (**B**). Residues that contributed substantially to interactions are colored as in Fig. 3. Mutations that were introduced are marked in bold. Immunity protein and colicin/pyocin positions are numbered according to Im7 (Im7#) and Col-E7 (E7#) and, together with the Im7/E7 secondary structure elements, are shown above and below the alignment.

Comparing the energy contributions in each of these complexes, we observed substantial differences in the identity and contributions of individual residues relative to the respective wild-type structures (Fig. 9). The first computationally redesigned Col-E7–Im7 complex incorporated one mutation in the α2 helix of Im7 and two mutations in the α4–β2 loop of Col-E7^45^. A follow-up study incorporated an additional substitution in the α2–α3 loop of Im7 and additional two mutations in the α4 helix and α4–β2 loop of Col-E7^46^. Interestingly, in the more extensively redesigned Im7, four residues in the α1–α2 motif lost their electrostatic contributions, even though the substituted residues were in the α2 helix and α2–α3 loop (Fig. 9A). In the redesigned E7 colicins, however, all differences in contributions were observed in the mutated residues or in adjacent residues, most of which involved a loss of electrostatic contribution as a direct result of the change in physico-chemical properties of the substituted residue. For example, in both computationally-redesigned complexes, mutations of Im7-Asp35 to a tyrosine and Col-E7-Lys528 to a glutamine removed the salt bridge between these two residues, and concurrently, their calculated electrostatic contributions.

In the Col-E9–Im2 non-cognate complex, most of the contributions on either side of the interface were identical to those seen in the respective cognate complexes, with the exception of the Im2 α2 helix (Fig. 9A). In this helix, the pattern of Im2 contributions in the non-cognate complex was the same as the contributions of Im9 in its cognate complex with Col-E9 (Fig. 9A). The three electrostatic contributions in the α2 helix that were observed only in the Col-E2–Im2 cognate complex depend on three residues that are unique to Col-E2 (Fig. 6).

In the two in vitro evolved Im9 proteins, the introduction of 7 or 9 mutations, spread across the entire interface, resulted in a novel and dramatically dissimilar contribution pattern from the α1–α2 motif. In contrast to the cognate complexes of Col-E7–Im7 and Col-E9–Im9, most of the electrostatic contributions from the evolved Im9 α1–α2 motif were only main chain electrostatic contributions. Surprisingly, the contributions from the α2–α3 region of the evolved Im9 proteins resembled those of wild-type Im9, rather than wild-type Im7 (Fig. 9A), suggesting the α1–α2 motif has a dominant role in determining specificity in these pairs.

Finally, we analyzed two designed Col-E2–Im2 complexes, where the Im2 α1–α2 loops were replaced with computationally-designed loops, complemented with mutations in Col-E2 to increase affinity^48^. One designed immunity protein (Im-des3, which forms a “specific pair”) exhibited relatively high specificity to its cognate redesigned Col-E2, while the second immunity protein (Im-des7, which can form alternate “multi-specific pairs”) exhibited high affinity for multiple designed Col-E2 proteins. We observed a dramatic reduction of electrostatic contributions in both designed immunity proteins, compared to the wild type Im2–Col-E2 complex (Fig. 9A), supporting the hypothesis that abundant electrostatic interactions maintain high specificity. On the colicin side of the designed interfaces, we observed a smaller yet distinct reduction in electrostatic contributions (Fig. 9B). Strikingly, the α1–α2 motif in Im2-des3, which forms a specific pair, lost all three of its main chain electrostatic contributions, but three new main chain electrostatic contributions are seen in the α2 helix and the α2–α3 loop (Fig. 9A). In the α2-α3 region, we observed two or four side chain electrostatic contributions in the Im2-des3 and Im2-des7, respectively, compared to 11 such contributions in the wild-type Im2. Generally, all electrostatic contributions observed from Im2-des3 and Im2-des7 were also present in the wild-type Im2–Col-E2 complex (Fig. 9A). On the other hand, we can attribute the observed losses of electrostatic contributions directly to the mutagenesis of the designed proteins. For example, in the specific Im-des3, an asparagine to isoleucine mutation (in the Im7#35 position) removed a side-chain to side-chain electrostatic interaction with a threonine residue in the E7#539 position, while the corresponding asparagine to glutamine mutation in the multi-specific Im2-des7 maintained this interaction. A second example is the loss of electrostatic contributions from two Im2-des3 aspartate residues (Im7#52 and Im7#63, Fig. 9A), due to a lysine to glycine mutation in position E7#518 and a lysine to glutamine mutation in position E7#531 (Fig. 9B).

### Residue-level mapping of Col-E5, Col-D, and Col-E3 with their cognate immunity proteins

**Figure 10:**
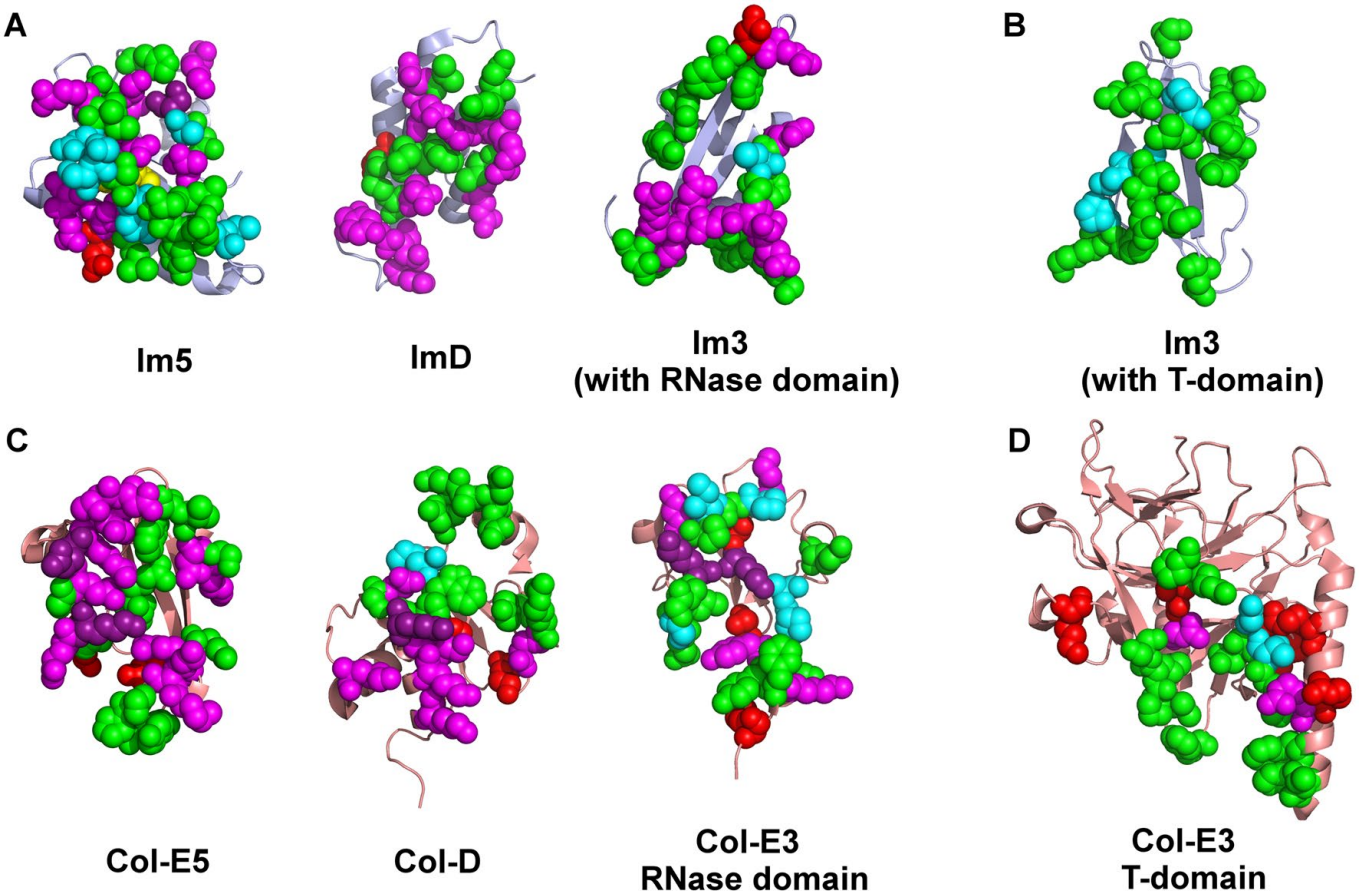
3D visulization of residues that substantially contribute to interactions between colicins E5, D, E3 and their cognate immunity proteins. (**A**) Residues in Im5, ImD, and Im3 that substantially contribute to interactions with their cognate partners. The three interfaces correspond to immunity protein interactions with the cytotoxic domains of the cognate colicins, similar to the compelxes analyzed in Fig. 4. (**B**) Residues in Im3 that substantially contribute to interactions with the translocation domain (T-domain) of Col-E3. This domain was truncated in the other structures analyzed here. (**C**) Residues in colicins E5, D and E3 that substantially contribute to interactions with their cognate partners. Only the cytotoxic RNase domains are shown. (**D**) Residues in the T-domain of Col-E3 that substantially contribute to interactions with Im3. Immunity proteins and colicins/pyocins are shown as ribbons colored light blue and salmon, respectively. The results were visualized on the following structures: Col-E5–Im5 (2FHZ), Col-D–ImD (1V74), Col-E–Im3 (2B5U). All contributing residues are shown as spheres, colored as in Fig. 4.

We also applied our energy-based computational methodology to the complexes we classified as structurally-dissimilar from the E7/Im7-like families (Fig. 1 B-D): Col-E5–Im5, Col-D–ImD, and Col-E3–Im3 (Fig. 10). The Col-E5–Im5 interface was substantially larger than all other complexes—our calculations identified 28 Im5 residues that contribute significantly to interactions with Col-E5. However, in ImD and in each of the two separate interfaces of Im3, there were 15 to 20 contributing residues, similar to the interface of the Im7-like family with their colicin partners (Fig. 10A vs. Fig. 3A). Also similar to the Im7-like family, in Im5, ImD, and the region of Im3 that interacts with the RNase domain of Col-E3, most of the contributing residues did so via electrostatic interactions (Fig. 10A). In contrast, in the region of Im3 that binds to the translocation domain of Col-E3, most of the contributing residues did so only via non-polar interactions (Fig. 10B). On the colicin side of the interfaces, Col-E5 had 27 contributing residues, while Col-D and either of the two domains of Col-E3 had 18 to 22 contributing residues, similar to the E7-like family members (Fig. 10B vs. Fig. 3B).

## Discussion

Using continuum electrostatic and burial-based energy calculations, we mapped the interactions of all representative colicin/pyocin with immunity proteins, providing a comprehensive and quantitative comparison of these complexes at the individual residue level. On both sides of the interfaces between colicins/pyocins and their immunity protein partners, our calculations show that almost every residue in the interface or in its vicinity contributes to intermolecular interactions. Most of these residues show both non-polar and electrostatic contributions and more than a third of these contributions were not observed in previous studies. We note that one of the advantages of our computational approach is the ability to accurately detect electrostatic contributions that contribute favorably to binding, and, uniquely, also those coming from main-chain atoms. Indeed, our computational analysis indicates that while the global geometry and non-polar contributions in the cognate complexes of the E7/Im7-like families were similar, a striking diversity in electrostatic interactions among these complexes underlies their specific interactions. A similar central role of electrostatic/polar interactions in determining either affinity or specificity was also observed previously in other protein–protein interfaces, albeit usually involving a less extensive network of interactions^62–64^. Our results therefore suggest that evolution combined a “contribution-rich” interface with abundant and specific electrostatic interactions to achieve both ultra-high affinity and exquisite specificity between different colicins and immunity proteins.

Our analysis showed here, for the first time, that the α1–α2 motif in immunity-proteins is a major specificity determinant towards colicins/pyocins, not only in engineered Im7-like family members, as noted previously^47,48,55^, but notably also in all wild type immunity proteins. While Levin et al*.* interpreted the effect of mutating residues in the α1–α2 motif to reach successful engineered complexes as an indirect effect on intermolecular interactions, our analysis uncovered numerous direct contributions from residues in this motif. Unexpectedly, our calculations show that about half of the residues across the various α1–α2 motifs contribute significantly to intermolecular interactions, mostly via electrostatic contributions, with many of the latter contributions originating from the main chain of the residues. The latter observation may explain why only a small minority of these contributing residues were identified in previous studies as residues that interact directly with colicin/pyocins^14,26,36–38,40,47^. We also note that in most of these studies, the only α1–α2 residues that were identified were the two adjacent acidic residues at the C-terminal end of the α1–α2 motif. Moreover, our comparative analysis suggests that the α1–α2 motif plays a general and dominant role in determining specificity through a combination of dissimilar motif geometry and divergent electrostatic contributions. A striking exception is the α1–α2 motifs in Im2 and Im9, which are structurally similar and exhibit a similar pattern of electrostatic contributions in both cognate and non-cognate complexes. Indeed, substitution of the Im2 α2 helix with its Im9 counterpart was sufficient to increase binding affinity towards Col-E9 to the level of the cognate complexes^41^, showing the α1–α2 motifs of Im2 and Im9 are indeed interchangeable. Our suggestion for a general role of the α1–α2 motif as a major specificity determinant among the wild-type proteins is also supported by the necessity to substitute the α1–α2 motif with a novel amino acid sequence in the in vitro evolved Im9^47^ and in the computationally redesigned Im2 proteins^48^. Notably, even though these engineered α1–α2 motifs were dissimilar to their wild-type counterparts, in all of these proteins we observed contributions from the majority of the α1–α2 motif residues, emphasizing its importance. Lastly, this region was shown to be particularly divergent in sequence and length across a larger set of 17 immunity protein homologous to the IM7-like family members^14^, suggesting the α1–α2 motif functions as a major specificity determinant across most or all of these proteins.

Our analysis also showed that electrostatic interactions play a key role in setting dissimilar interactions across the interface between colicins/pyocins and their immunity proteins. This implies that the α1–α2 motif and electrostatic/polar interactions across the rest of the interface set interaction specificity together. Surprisingly, the colicin residues that interact with the structurally-dissimilar α1–α2 motif showed a similar pattern of polar/electrostatic contributions. On the other hand, diverse electrostatic contributions from colicin residues were observed in the interactions with the more conserved immunity protein α3 helix, suggesting that colicin interactions with the Im α3 region also contribute to interaction specificity at the family level. Therefore, the more divergent parts in the immunity proteins interact with more conserved parts in colicins/pyocins, and vice versa.

Our computational analysis identified 11 to 17 contributing residues in the Im α2–α3 region—the majority of contributing residues across the immunity proteins. While many of these residues (~ 70%) were listed in some previous structural studies of Im7, ImS2, and ImAP41 as potential contributors to intermolecular interactions^14,38^, to date no mutagenesis studies have been performed on these immunity proteins. However, previous studies did mutate 8 out of our 12 predicted Im9 contributions and 6 out of our 14 predicted Im2 residues in the α2–α3 region, all leading to a substantial reduction in binding affinity^26,40^—supporting the accuracy of our predictions. On the other hand, four and seven of our predicted contributing residues in Im9 and Im2, respectively, were also not tested in previous experimental studies—most of these contribute via electrostatic interactions. On the other side of the interface, we identified 15 to 20 colicin/pyocin contributing residues, mostly in the α4–β4 region. Previous experimental data on this side of the interface is scarce, as most colicins and pyocins were not investigated biochemically at the individual residue level. Only some Col-E9 residues were mutated in a previous study, and these results indeed validated our predictions for six contributing residues^29^. Therefore, our residue-level maps can guide further biochemical studies of these interactions.

In summary, the energy-based computational analysis described here offers a quantitative framework that enables comparing, at the individual residue level, wild-type, engineered, and non-cognate complexes of colicins/pyocins with immunity proteins. Indeed, we also applied our approach to the complexes of Col-E3–Im3, Col-E5–Im5, and Col-D–ImD and observed that these pairs exhibit a divergent pattern of contributions, yet also exhibit a dominant role of electrostatics in determining intermolecular interactions. Since all of the structures we analyzed belong to larger families that can be well-aligned at the sequence level^14,22^, our results can be directly extrapolated to a much wider dataset of colicins and pyocins and their respective immunity proteins, In a wider perspective, our results provide residue-level insights into a model system of protein–protein interactions at the family level, show how electrostatics interactions play a role in determining specificity at the family level, and detail precise residue-level information that can be used for directed therapeutic interventions.

## Materials and methods

### Protein structures

The following representative 3D structures were used in our analysis and visualization of colicin/pyocin–immunity protein complexes (with PDB IDs): Col-E7–Im7 (7CEI, 2JAZ, 2JB0, 2JBG, 1ZNV)^38,65–67^, computational redesigned complexes of Col-E7–Im7 (1UJZ, 2ERH)^45,46^, complexes of in vitro evolved Im9 with Col-E7 (3GJN, 3GKL)^47^, Col-E9–Im9 (1EMV)^36^, Col-E2–Im2 (3U43)^37^, complexes of rationally-redesigned Col-E2–Im2 (6ERE, 6ER6)^48^, the non-cognate complex of Col-E9–Im2 (2WPT)^30^, Pyo-S2–ImS2 (4QKO)^14^, Pyo-AP41–ImAP41 (4UHP)^14^, Col-E3–Im3 (2B5U)^49^, Col-E5–Im5 (2DFX, 2FHZ)^52,54^, Col-D–ImD (1V74, 1TFK)^50,51^. Hydrogen atoms were added using CHARMM and the structures were subjected to conjugate gradient minimization with a harmonic restraint force of 50 kcal/mol/Å^2^ applied to the heavy atoms. Structure alignments were performed using the Combinatorial Extension (CE) method, as implemented in the RCSB protein comparison tools (https://www.rcsb.org/pdb/workbench/workbench.do). 3D structural visualizations were carried out with the PyMol molecular graphics program (https://www.pymol.org/).

### Energy calculations to map residue-level specificity determinants

We followed the methodology described previously^55–61,68^ to analyze the per-residue contributions of residues in colicins/pyocins and immunity proteins to interactions with their partners in the crystal structures mentioned above. The Finite Difference Poisson–Boltzmann (FDPB) method, as implemented in DelPhi^69^, was used to calculate the net electrostatic/polar contributions (ΔΔG_elec_) of each residue found within 15 Å of the dimer interface in each complex. For each residue, electrostatic contributions from each side chain or the entire residue were calculated separately (see Supplementary Figs. S1–S3) and comparison of these separate calculations was used to determine if electrostatic contributions originate from the side-chain of a residue, the main chain, or both. Residues contributing ΔΔG_elec_ ≥ 1 kcal/mol to the interactions (twice the numerical error of the electrostatic calculations) were deemed as substantially contributing to the interactions^55^. Non-polar energy contributions (ΔΔG_np_) were calculated as a surface-area proportional term by multiplying the per-residue surface area buried upon complex formation, calculated using Surfv^70^, by a surface tension constant of 0.05 kcal/mol/Å^2^. Residues contributing ΔΔG_np_ ≥ 0.5 kcal/mol to the interactions (namely, those that bury more than 10 Å^2^ of each protein surface upon complex formation) were defined as making substantial non-polar contributions. Note that according to these definitions, partially buried residues that make a substantial non-polar contribution to binding are often also partially exposed to the solvent. On the other hand, residues that make substantial electrostatic contributions can either also make non-polar contributions (i.e., be partially or fully buried in the interface) or contribute only via long-distance electrostatic interactions. To reduce false positives and negatives, we applied a consensus approach across comparable biological replicates in multiple PDB structures (7CEI, 2JAZ, 2JB0, 2JBG, 1ZNV—see Supplementary Fig. S1; 2DFX, 2FHZ; 1V74, 1TFK) or across multiple dimers in an asymmetric unit (4QKO; 4UHP), substantially improving prediction accuracy.

## Supplementary Information

Supplementary Information.
