# Supplementary material for: Structural design principles for specific ultra-high affinity interactions between colicins/pyocins and immunity proteins
Source: Sci Rep. 2021 Feb 15;11:3789. doi: 10.1038/s41598-021-83265-2 (PMC7884437; doi:10.1038/s41598-021-83265-2)
Supplement: Supplementary file 1 — Supplementary Information. [file 41598_2021_83265_MOESM1_ESM.pdf]

# Supplementary information

## Structural design principles for specific ultra-high-affinity interactions between colicins/pyocins and immunity proteins

Avital Shushan and Mickey Kosloff

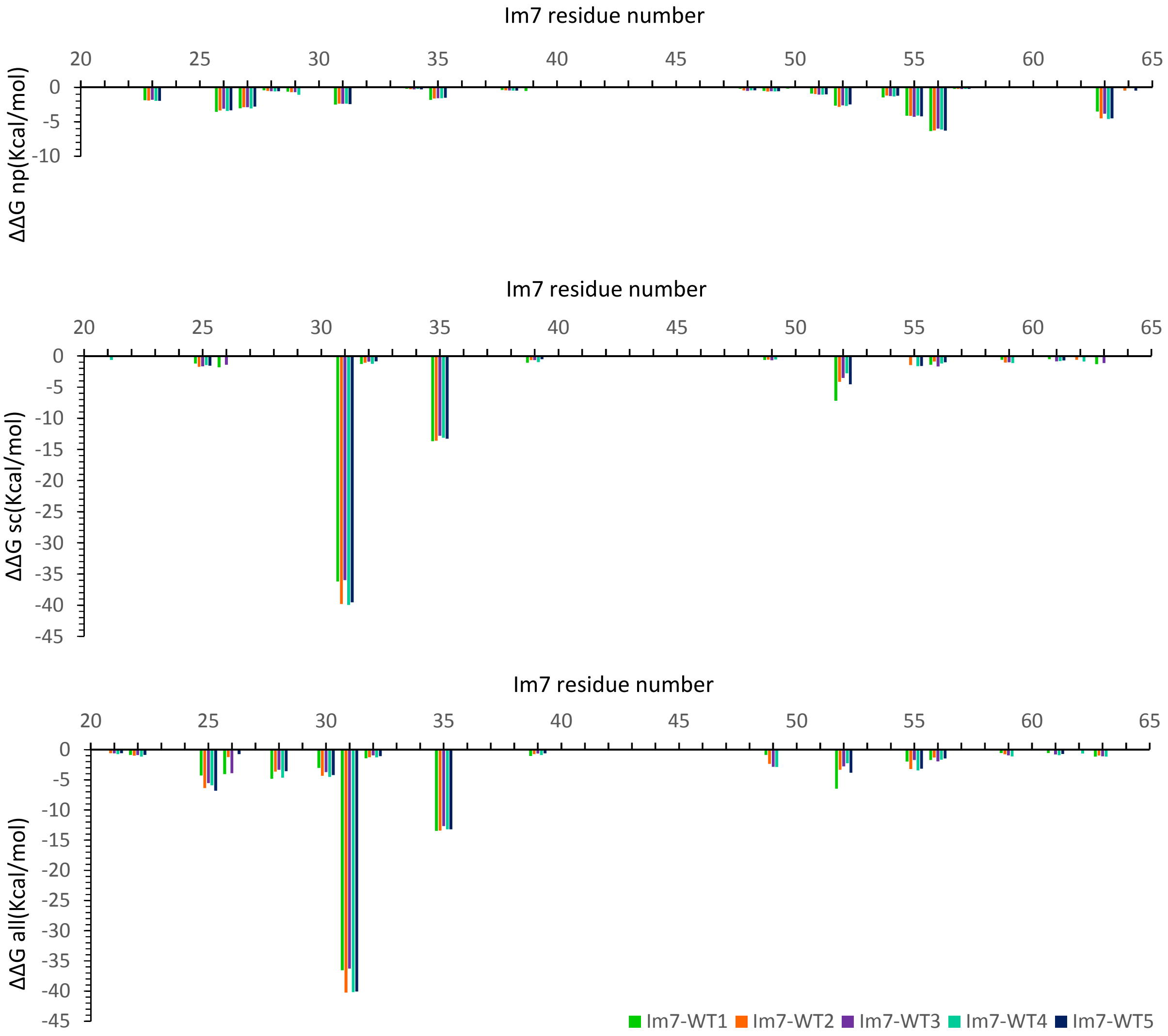


**Supplementary Figure S1. IM7 per-residue energy contributions to interactions with Col-E7 in five comparable biological replicates from different X-ray structures**. Panels show the results of the energy calculations for the interactions of five biological replicates of wild type complexes of Col-E7–IM7, as described in the Methods, for PDB IDs: 7CEI, 2JAZ, 2JB0, 2JBG, and 2ZNV.

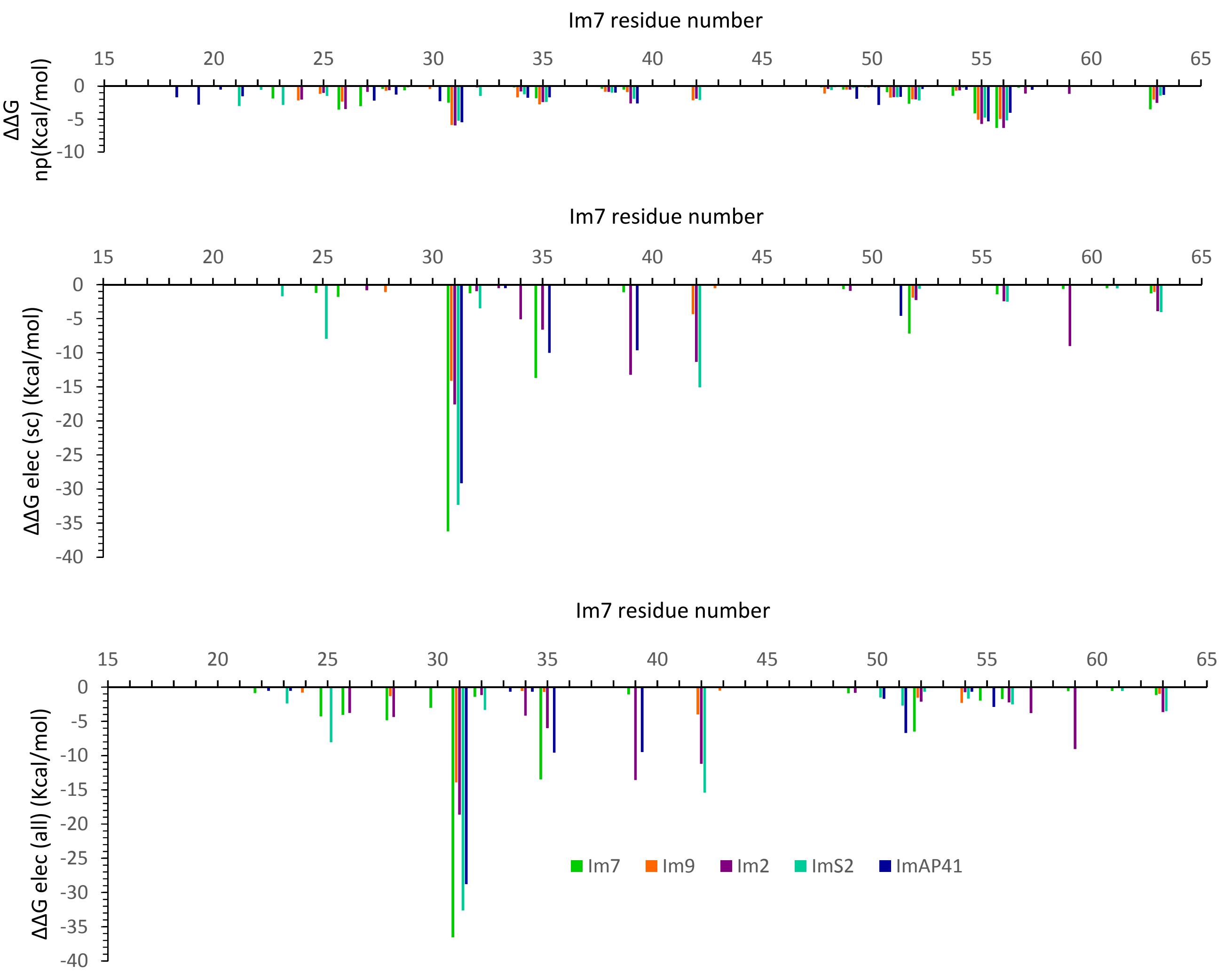


**Supplementary Figure S2. Per-residue energy contributions to interactions of immunity proteins from the Im7-like family with their cognate colicins/pyocins**. Panels show the results of the energy calculations for the interactions of Im7, Im9, Im2, ImS2, and ImAP41 with their cognate colicins, as described in the Methods for PDB IDs: 7CEI, 1EMV, 3U43, 4QKO, and 4UHP. Immunity proteins were aligned to Im7, as in Fig. 3A.

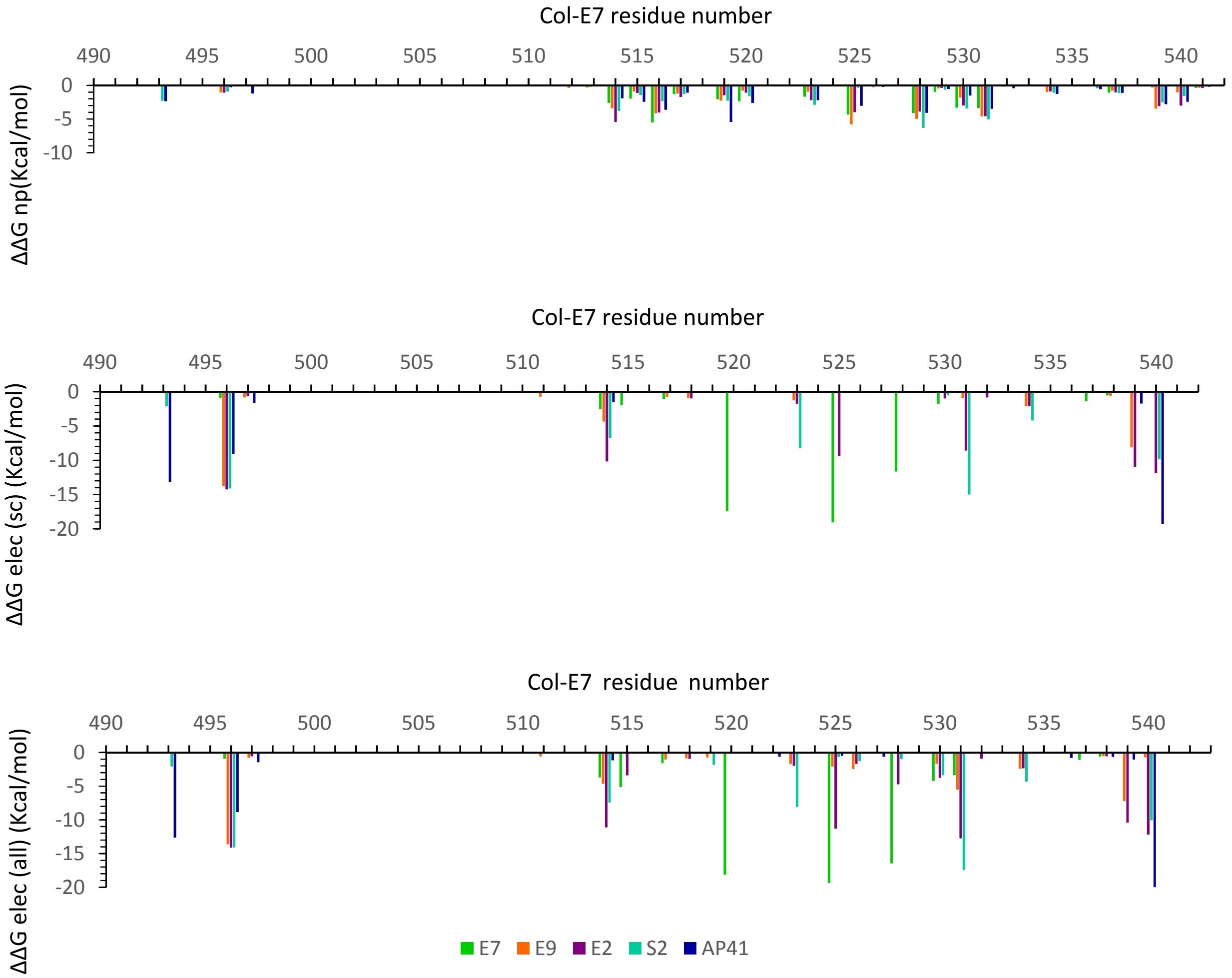


**Supplementary Figure S3. Per-residue energy contributions to interactions of different colicins/pyocins with their cognate immunity proteins**. Panels show the results of the energy calculations for the interactions of colicins/pyocins E7, E9, E2, S2, and AP41 with their cognate immunity proteins, calculated as described in Methods for PDB IDs: 7CEI, 1EMV,3U43, 4QKO, and 4UHP.

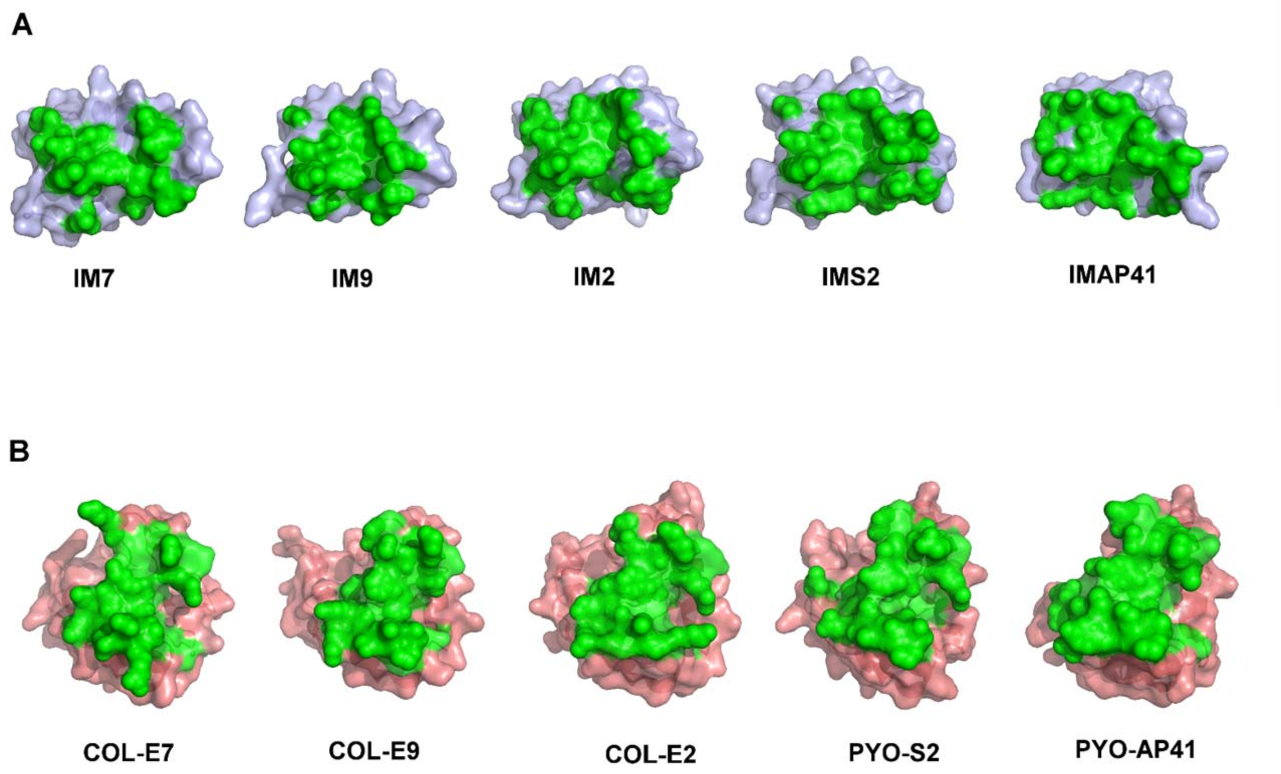


**Supplementary Figure S4. Similar non-polar contributions across different colicin/pyocin–immunity protein complexes. A.** Immunity protein residues that are part of the interface with their cognate colicins/pyocins. **B.** Colicin/pyocin residues that are part of the interface with their cognate immunity proteins. Immunity proteins and colicins are shown in molecular surface representations colored salmon and light blue, respectively. Residues with non-polar contributions in the complex (i.e., part of the interface) were defined as those with ≥ 1 $Å^2$ of their surface buried upon complex formation and are colored green.

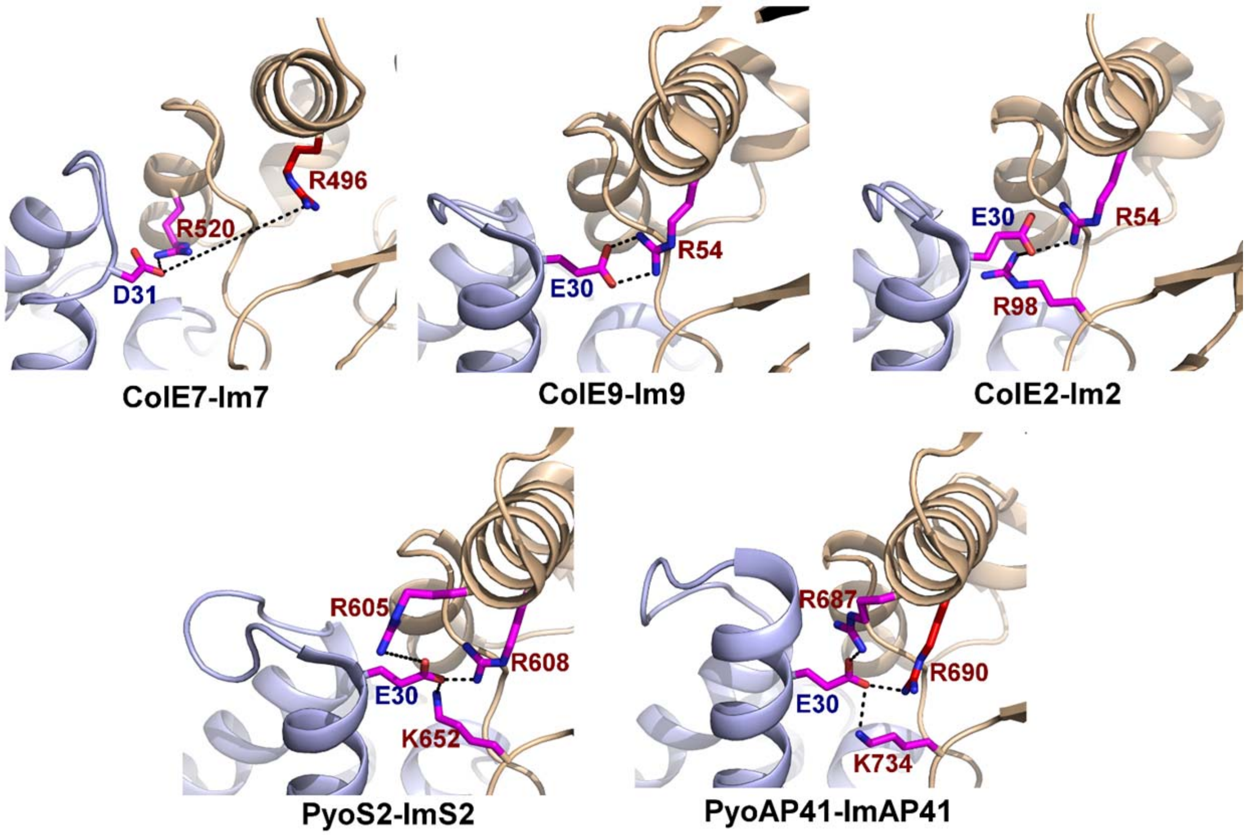


**Supplementary Figure S5. Converging positively-charged colicin/pyocin residues interact similarly with a negatively-charged residue at the C-terminus of the Im α1-α2 motif.** Positively-charged colicin/pyocin residues from different structural elements (a lysine or arginine from either position E7#520 or E7#540 that reach a similar orientation and arginines from positions E7#493 and E7#496, labeled in dark red) converge similarly in all complexes to interact with a negatively-charged immunity protein residue (Im7#31), labeled in blue. Immunity proteins and colicins/pyocins are shown as ribbons, colored light blue and wheat, respectively. Substantially contributing residues are shown as sticks, colored as in Fig. 6. Electrostatic interactions are marked with dashed black lines.

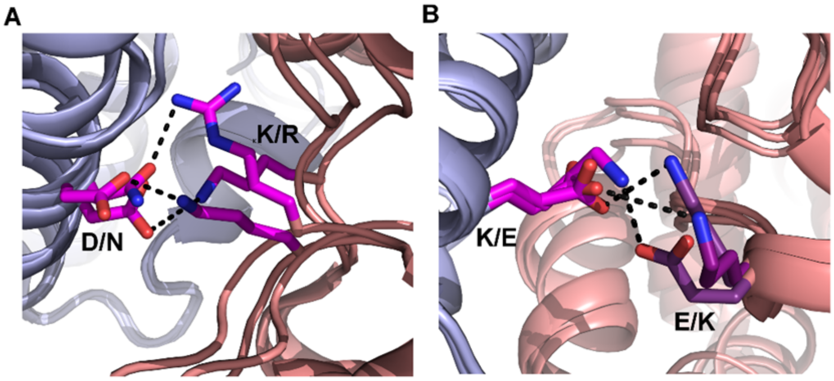


**Supplementary Figure S6. Similar electrostatic interactions between converging colicin/pyocin residues and residues in the Im α2 region. A.** A positively-charged lysine/arginine residue ("K/R", E7#528/E7#540) – K528 in Col-E7, R98 in Col-E2, and K734 in Pyo-AP41 – interacts similarly with an aspartate/asparagine residue ("D/N", Im7#35) in the Im α2 helix. **B.** A salt bridge between a lysine (K89) in Col-E9 and Col-E2 or a glutamate (E643) in Pyo-S2 ("K/E", E7#531) and a glutamate in Im9/Im2 (E41) or a lysine (K42) in Im-S2, which are located in the Im α2 helix ("E/K", Im7#42). Immunity proteins and colicins/pyocins are shown as ribbons colored light blue and salmon, respectively. Substantially contributing residues are shown as sticks, colored as in Fig. 6. Electrostatic interactions are marked with dashed black lines.

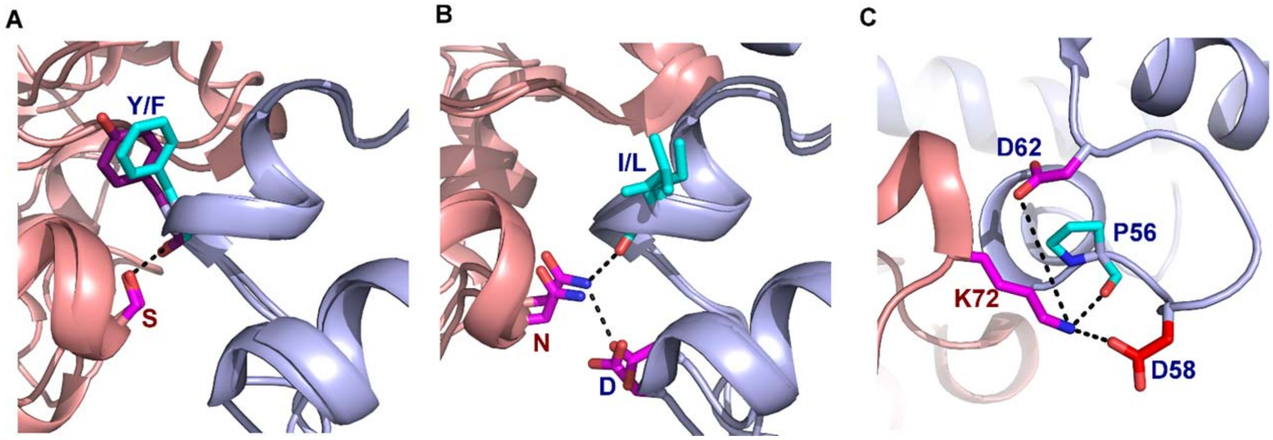


**Supplementary Figure S7. Divergent electrostatic interactions of residues in position E7#514 with residues in the Im α3 region. A.** In Col-E7–Im7 and PyoAP41–ImAP41, a serine residue in position E7#514 (“S”) interacts with a tyrosine/phenylalanine residue (“Y/F”, Im7#55). **B.** In Col-E9 and Pyo-S2, an asparagine residue in position E7#514 (“N”) interacts with isoleucine/leucine and aspartate residues (“I/L” and “D”, Im7#54 and Im7#63). **C.** A Col-E2 lysine in position E7#514 (K72) interacts with two aspartate residues (Im2 D58 and D62) and with a proline residue (Im2 P56). The angle of view is rotated 90° about the X-axis and 20° about the Y-axis relative to A and B. Immunity proteins and colicins/pyocins are shown as ribbons colored light blue and salmon, respectively, with their residues labeled in blue and dark red, respectively. Substantially contributing residues are shown as sticks, colored as in Fig. 6. Electrostatic interactions are marked with dashed black lines.
